# Antibiotics or probiotics as preventive measures against ventilator-associated pneumonia: a literature review

**DOI:** 10.1186/cc9963

**Published:** 2011-01-13

**Authors:** Marcus J Schultz, Lenneke E Haas

**Affiliations:** 1 Department of Intensive Care Medicine, Academic Medical Center, University of Amsterdam, Meibergdreef 9, 1105 AZ Amsterdam, The Netherlands; 2 Laboratory for Experimental Intensive Care and Anesthesiology, Academic Medical Center, University of Amsterdam, Meibergdreef 9, 1105 AZ Amsterdam, The Netherlands; 3 HERMES Critical Care Group, Academic Medical Center, University of Amsterdam, Meibergdreef 9, 1105 AZ Amsterdam, The Netherlands

## Abstract

**Introduction:**

Mechanically ventilated critically ill patients frequently develop ventilator-associated pneumonia (VAP), a life-threatening complication. Proposed preventive measures against VAP include, but are not restricted to, selective decontamination of the digestive tract (SDD), selective oropharyngeal decontamination (SOD) and the use of probiotics. Probiotics are live bacteria that could have beneficial effects on the host by altering gastrointestinal flora. Similar to SDD and SOD, a prescription of probiotics aims at the prevention of secondary colonization of the upper and/or lower digestive tract.

**Methods:**

We performed a literature review to describe the differences and similarities between SDD/SOD and probiotic preventive strategies, focusing on (a) efficacy, (b) risks, and (c) the routing of these strategies.

**Results:**

Reductions in the incidence of VAP have been achieved with SDD and SOD. Two large randomized controlled trials even showed reduced mortality with these preventive strategies. Randomized controlled trials of probiotic strategies also showed a reduction of the incidence of VAP, but trials were too small to draw firm conclusions. Preventive strategies with antibiotics and probiotics may be limited due to the risk of emerging resistance to the locally applied antibiotics and the risk of probiotic-related infections, respectively. The majority of trials of SDD and SOD did not exhaustively address the issue of emerging resistance. Likewise, trials of probiotic strategies did not adequately address the risk of colonization with probiotics and probiotic-related infection. In studies of SDD and SOD the preventive strategy aimed at decontamination of the oral cavity, throat, stomach and intestines, and the oral cavity and throat, respectively. In the vast majority of studies of probiotic therapy the preventive strategy aimed at decontamination of the stomach and intestines.

**Conclusions:**

Prophylactic use of antibiotics in critically ill patients is effective in reducing the incidence of VAP. Probiotic strategies deserve consideration in future well-powered trials. Future studies are needed to determine if preventive antibiotic and probiotic strategies are safe with regard to development of antibiotic resistance and probiotic infections. It should be determined whether the efficacy of probiotics improves when these agents are provided to the mouth and the intestines simultaneously.

## Introduction

Ventilator-associated pneumonia (VAP) frequently complicates the course of intubated and mechanically ventilated critically ill patients [1-3]. VAP is associated with a decreased survival [4], although it is difficult to quantify the exact attributable mortality [5,6]. Several approaches for the prevention of VAP have been proposed, including the use of ventilator bundles, specific practical measures such as hand hygiene in healthcare workers, isolated interventions to prevent tracheal aspiration, such as semi-recumbent positioning and subglottic aspiration, and the use of silver-coated tubes [7-10].

Prevention of colonization of the upper and/or lower digestive tract is another approach for the prevention of VAP. This approach is built on the theory that the gastrointestinal flora changes with acute illness. In particular, it assumes that the normal flora disappears and is replaced by an overgrowth of so-called potentially pathogenic microorganisms (PPM), followed by aspiration of PPM, which could finally result in VAP.

There are roughly two approaches for the prevention of colonization of the upper and/or lower digestive tract. One strategy includes topical application of non-absorbable antibiotics. Prevention of VAP has been achieved in trials of selective decontamination of the digestive tract (SDD) and trials of selective oropharyngeal decontamination (SOD). Another strategy uses topically applied probiotics, live bacteria that could alter gastrointestinal flora. Recent trials of different probiotic formulas suggest this strategy also to be effective in the prevention of VAP.

This manuscript describes the rationale behind prophylactic antibiotic and probiotic strategies in critically ill patients. This is followed by a review dealing with the beneficial effects, risks, and routing of prophylactic antibiotic or probiotic therapy. This manuscript does not deal with oropharyngeal decontamination with chlorhexidine, which has the same principles as SOD. Isolated interventions for the prevention of tracheal aspiration are also not discussed.

## Materials and methods

### Data sources

Two methods were used to identify relevant manuscripts in the medical literature on SDD, SOD and probiotic (or synbiotic) strategies. First, an electronic search in the databases of Medline, Embase, the Cochrane Library, the Cochrane Database of Systematic Reviews and Sumsearch was conducted. Second, reference lists of identified and selected manuscripts were reviewed for additional relevant manuscripts. The search was restricted to manuscripts published from 1980 until now, and manuscripts written in English.

### Keywords (MeSH and text word)

The following keywords were used to identity relevant manuscripts: "critical care", "intensive care", "ventilator-associated pneumonia", "nosocomial pneumonia", "SDD", "selective decontamination of the digestive tract", "selective gut decontamination", "SOD", selective oropharyngeal decontamination", "synbiotic", "prebiotic", and "probiotic".

### Study selection

Titles and abstracts of identified manuscripts were reviewed on: a) population (that is, adults in and type of intensive care unit), b) intervention (that is, SDD, SOD or probiotic therapy), c) outcome (VAP and mortality), and d) type of study (randomized controlled trial or other study types). In case of uncertainty the complete manuscript was obtained and evaluated. We did not restrict inclusion of manuscripts on methodological quality or any other critically appraisal criteria other than the criteria we formulated for data extraction. We restricted inclusion of manuscripts of SDD to those studies that evaluated an SDD-regimen consisting of administration of non-absorbable antibiotics in the mouth *and *intestines, *and *a short course of systemic antibiotics. We restricted inclusion of manuscripts of SOD to those studies that evaluated an SOD-regimen consisting of the administration of non-absorbable antibiotics *solely *in the mouth. We included all manuscripts of probiotic therapy, (that is, administration of probiotics could be in the mouth, *or *the intestines, *or *both).

Finally, we restricted inclusion of manuscripts to those that dealt with the general ICU population (that is, studies in highly specific patient groups, such as liver transplant patients, and studies of pediatric patients were ignored).

### Data extraction

Manuscripts were criticized along three subjects: 1) Is prophylactic use of antibiotics or probiotics preventing VAP and reducing mortality? 2) What are the risks of preventive use of antibiotics or probiotics in critically ill patients? 3) What is the optimal route of administration of preventive antibiotics or probiotics?

## Results

### The rationale for antibiotics or probiotics as preventive measures against infections

#### Critical illness-associated infections

Critical illness-associated infections have been hypothesized to be either primary endogenous or secondary endogenous in their origin [11]. In this theory, primary endogenous infections are caused by pathogens carried in the oral cavity, throat, stomach and/or intestines of patients on admission to the ICU. Secondary endogenous infections are caused by pathogens thought to be absent in the upper and lower digestive tract on admission, but to be acquired during the stay in ICU. A short course of system antibiotics would prevent primary endogenous infections. Secondary endogenous infections would be banned if colonization could be prevented.

A second theory concerns the pathogenicity of microorganisms [11]. Pathogenicity can be expressed in the "Intrinsic Pathogenicity Index" (IPI), the number of patients infected by species X divided by the number of patients carrying species X in the oropharynx, stomach and/or intestines. Theoretically, the range of the IPI is 0 to 1: carriage of a microorganism with an IPI close to 0 would seldom be followed by an infection; carriage of a microorganism with an IPI close to 1 would almost always be followed by an infection. Prevention of carriage with pathogens with an IPI close to 1 would benefit critically ill patients, by preventing infections.

In addition, disturbance or loss of the intact anaerobic intestinal flora have been hypothesized to increase colonization with subsequent higher infection rates [12]. Disturbance or loss of the anaerobic flora would lead to increased colonization and increased infection risk with facultative aerobic bacteria. In this theory, it has been suggested that most of the infections in ICU patients are preceded by colonization of the stomach and intestines with pathogenic micro-organisms.

#### SDD and SOD

SDD consists of selective eradication of PPM in the oral cavity and decontamination of the stomach and intestines by local administration of non-absorbable antibiotics, - the first is reached by application of a paste, gel or lozenge to the oral cavity, the second by administration of a suspension through a nasogastric tube. Systemic prophylaxis is provided by a short course of an intravenous antimicrobial agent, to prevent respiratory infections caused by commensal respiratory flora. Notably, the classical design of SDD also includes hand hygiene by health care workers, and frequent surveillance cultures.

SOD consists of selective eradication of PPM in the oral cavity by local administration of non-absorbable antibiotics. SOD has been combined inconsistently with systemic prophylaxis by a short course of an intravenous antimicrobial agent.

#### Probiotics

The concept of selective decontamination with probiotics, with or without prebiotics, is at least in part based on colonization resistance. Probiotics are live bacteria that could have a beneficial effect on the host by altering gastrointestinal flora. Prebiotics are non-digestible sugars that selectively stimulate the growth of certain colonic bacteria. When administered in combination, prebiotics could enhance the survival of probiotic strains as well as stimulate the activity of the endogenous flora. The combination of pre- and probiotics has been termed "synbiotics".

Administration of probiotics is not expected to eradicate the PPM as antibiotics would do, but delaying the time to colonization while the patients are intubated and ventilated could be beneficial. Several probiotic and synbiotic formulas are known and used. They usually are a combination of lactic acid bacteria (including *Lactobacillus *spp.) plus prebiotics, or a single-agent probiotic (*Lactobacillus *spp.).

### Search results

The search recognized 64 manuscripts on SDD, 6 manuscripts on SOD and 9 manuscripts on probiotics. Additional relevant manuscripts were not found in the reference lists of identified and selected manuscripts. Thirty manuscripts potentially answered one or more of the above-mentioned questions.

#### Randomized controlled trials of prophylactic antibiotics

We identified 17 randomized controlled trials of SDD [13-29], 5 randomized controlled trials of SOD [30-34], and 8 randomized controlled trials of probiotics [35-42] with VAP as one of the endpoints in critically ill patients in general surgical and/or medical ICUs. Study details and the main results of trials of SDD, SOD and probiotics are presented in Tables 1, 2 and 3.

**Table 1:** Randomized controlled trials of selective decontamination of the digestive tract (SDD)^a,b^

Author	*n*	VAP incidence (versus control) - %	*P*-value	Mortality (versus control) - %	*P*-value
Kerver [13]	96	12 vs. 85%	< 0.001	29 vs. 32%	NS
Ledingham [14]	324	2 vs. 11%	0.006	24 vs. 24%	NS
Ulrich [15]	100	15 vs. 50%	< 0.001	31 vs. 54%	< 0.02
Aerdts [16]	88	0 vs. 26%	0.0001	12 vs. 15%	NS
Blair [17]	331	7 vs. 26%	0.002	15 vs. 19%	NS
Hartenauer [18]	200	10 vs. 45%	< 0.01	31 vs. 36%	NS
Gastinne [19]	445	12 vs. 15%	NS	34 vs. 30%	NS
Cockerill [20]	150	4 vs. 5%	NS	11 vs. 19%	NS
Hammond [21]	322	7 vs. 6%	NS	12 vs. 12%	NS
Jacobs [22]	91	0 vs. 9%	NS	39 vs. 54%	NS
Rocha [23]	101	15 vs. 46%	< 0.001	21 vs. 44%	< 0.05
Winter [24]	183	3 vs. 18%	< 0.05	36 vs. 43%	NS
Ferrer [25]	80	18 vs. 24%	NS	31 vs. 27%	NS
Palomar [26]	83	17 vs. 50%	0.005	24 vs. 31%	NS
Verwaest [27]	660	9 vs. 18%	0.026	18 vs. 17%	NS
Sánchez-García [28]	271	11 vs. 29%	< 0.001	39 vs. 47%	NS
Krueger [29]	546	2 vs. 11%	0.007	20 vs. 29%	NS

^a^Trials reporting incidence rates of pneumonia. ^b^Administration of non-absorbable antibiotics in the mouth *and *the intestines, combined with a short course of systemic antibiotics. VAP, ventilator-associated pneumonia; NS, not significant; -, no data available.

**Table 2:** Randomized controlled trials of selective oropharyngeal decontamination (SOD)^a,b^

Author	*n*	VAP incidence (versus control) - %	*P*-value	Mortality (versus control) - %	*P*-value
Rodriguiz-Roldan [30]	28	0 vs. 73%	< 0.001	30 vs. 33%	NS
Pugin [31]	52	16 vs. 78%	< 0.0001	6 vs. 28%	NS
Laggner [32]	67	3 vs. 12%	NS	27 vs. 41%	NS
Abele-Horn [33]	88	22 vs. 47%	< 0.05	19 vs. 17%	NS
Bergmans [34]	226	10 vs. 23%	0.04	29 vs. 43%	NS

^a^Trials reporting incidence rates of pneumonia. ^b^Administration of non-absorbable antibiotics solely in the mouth. VAP, ventilator-associated pneumonia; NS, not significant; -, no data available.

**Table 3:** Randomized controlled trials of probiotic therapy^a^

Author	*n*	VAP incidence (versus control) - %	*P*-value	Mortality (versus control) - %	*P*-value
Kotzampassi^b ^[35]	134	54 vs. 80%	0.03	14 vs. 30%	NS
Spindler-Vesel^b ^[36]	113	15 vs. 39%	0.03	8 vs. 6%	NS
Forestier^c ^[37]	236	24 vs. 23%	NS	-	-
Klarin^b ^[38]	50	4 vs. 14%	NS	22 vs. 19%	NS
Knight^b ^[39]	259	9 vs. 13%	NS	27 vs. 33%	NS
Morrow^d ^[40]	146	19 vs. 40%	0.007	18 vs. 21%	NS
Oudhuis^b,e ^[41]	348	15 vs. 21%	NS	26 vs. 26%	NS
Barraud^b ^[42]	167	26 vs. 19%	NS	25 vs. 24%	NS

^a^Trials reporting incidence rates of pneumonia. ^b^Administration of probiotics in the intestines. ^c^Administration probiotics in the mouth. ^d^Administration probiotics in the mouth *and *in the intestines. ^e^Probiotic therapy was compared with selective decontamination of the digestive tract. VAP, ventilator-associated pneumonia; NS, not significant; -, no data available.

SDD appears to be an effective preventive strategy against VAP (Table 1). Indeed, most studies showed reductions in the incidence of VAP with SDD [13-18,23,24,26-29]. Mortality, however, was affected in only two studies [15,23]. Notably, SDD regimens used were not always carefully described and concentrations and dosing frequencies varied. Also, feeding regimens and use of other antibiotics were described inconsistently. In addition, patient populations varied widely. It should also be noted that the diagnostic criteria for VAP were at times rather loose; investigators may very well have looked at the effect of SDD on bronchitis or maybe even only respiratory tract colonization, rather than VAP. Recent systematic reviews and meta-analyses, including the majority of trials found by us, confirmed SDD to be an effective strategy against VAP showing a reduced incidence of VAP [43-45].

SOD also appears to be an effective preventive strategy against VAP (Table 2). Four out of five studies showed reductions in the incidence of VAP with SOD [30,31,33,34]. Like SDD, SOD had no effect on mortality. Similar to the randomized controlled trials of SDD, studies of SOD were heterogeneous in many aspects. A recent meta-analysis of trials of SOD showed this strategy did not reduce the incidence of VAP [46].

#### Randomized controlled trials of prophylactic probiotics

Prophylactic use of probiotics also seems an effective preventive strategy against VAP, albeit it to a lesser extent (Table 3). Three out of eight studies showed a significant reduction of VAP with probiotics [35,36,40]. Probiotics had no effect on mortality. Notably, two studies [41,42] were stopped prematurely after a study reporting increased mortality in critically ill pancreatitis patients receiving probiotics [47]. In most studies, probiotics were administered solely to the stomach [35,36,38,39,41,42], in one study [37] solely to the mouth, and in one study to the stomach and the mouth [40]. Studies of probiotics were also very heterogeneous. Two recent meta-analyses of trials of probiotics in critically ill patients [48,49], of which one directly focused on the effect of probiotics on VAP [48], drew different conclusions. One meta-analysis showed administration of probiotics to be associated with lower incidence of VAP than standard care [48], the other meta-analysis suggested that this prophylactic strategy conferred no benefit [49].

#### Risks of prophylactic use of antibiotics in critically ill patients

One concern with prophylactic use of antibiotics is the risk of the emergence of resistant bacteria [50,51]. Notably, colonization with resistant bacteria or an increase of super-infections was reported inconsistently in the randomized controlled trials of SDD or SOD. In fact, the majority of trials of SDD/SOD did not exhaustively address the issue of emerging resistance, as most were not specifically designed for this outcome.

One study of SDD that specifically addressed the issue of microbial resistance found no evidence for the selection of resistant bacteria in patients receiving prophylactic antibiotics [29]. This was confirmed in another report of long-term use of SDD [52]. Another large study found that resistance rates of Gram-negative bacteria were actually higher in the control population than in the SDD-treated population [53]. Interestingly, a reduction in the incidence of multi-resistant *Klebsiella *spp. was seen with prophylactic antibiotic use in three other studies [54-56].

However, more recently it was shown that both SDD and SOD markedly affect the bacterial ecology, with rising ceftazidime resistance prevalence rates in the respiratory tract during intervention and a considerable rebound effect of ceftazidime resistance in the intestinal tract after discontinuation of SDD [57].

Because SDD and SOD are not active against resistant Gram-positive bacteria, it may promote colonization with bacteria such as *Staphylococcus aureus *and *Entrococcus faecalis*. SDD promotes colonization with resistant Gram-positive bacteria [25,27,28,58,59]. Also, more cases of Gram-positive bacteremia occurred in SDD-treated patients [27]. It should be noted, though, that these trials were all performed in countries with high endemicity for Gram-positive bacteria. One study suggests that the addition of oral vancomycin to SDD could prevent colonization with resistant Gram-positive bacteria [60].

#### Risks of probiotic strategies in critically ill patients

One could expect that use of probiotics could cause diarrhea in critically ill patients. Three of the eight studies reported on the incidence of diarrhea [35,39,42]. In these trials, the numbers of patients with diarrhea was not different between patients who received probiotics and patients who did not.

Another concern with probiotics is colonization or overgrowth with lactic acid bacteria. Notably, with probiotics live bacteria are given to patients who could be immunoparalyzed because of their critical disease. Such patients could become colonized with probiotics, and eventually develop probiotic-related disease. One recent trial of probiotics in patients with pancreatitis was stopped because of increased mortality [61]. In this study, prophylaxis with probiotics was associated with increased bacterial translocation and enterocyte damage in patients with organ failure. Trials of probiotics against VAP published so far did not sufficiently look at this feared side-effect, although one report explicitly mentioned that bacteremia with probiotics was not found [42].

On a pre-specified subgroup analysis, Barraud *et al. *found a reduction of the 28-day mortality among severe sepsis patients treated with probiotics (odds ratio for death 0.38, 95% confidence interval 0.16 to 0.93) [42]. In contrast, probiotics were associated with a higher mortality rate in non-severe sepsis patients (odds ratio for death 3.09, 95% confidence interval 0.87 to 11.01). An explanation for the reduction of the 28-day mortality among severe sepsis patients may come from the fact that these patients were sicker than non-severe sepsis patients and a treatment effect may have been only apparent in these more severely ill patients. This should be confirmed by additional specific trials. But the investigators could not exclude a deleterious effect of probiotics on the less severely ill patients than those included in the severe sepsis subgroup, although it was not linked to probiotic-related disease, in particular infections.

#### Route of administration of prophylactic agents

**Figure 1:**
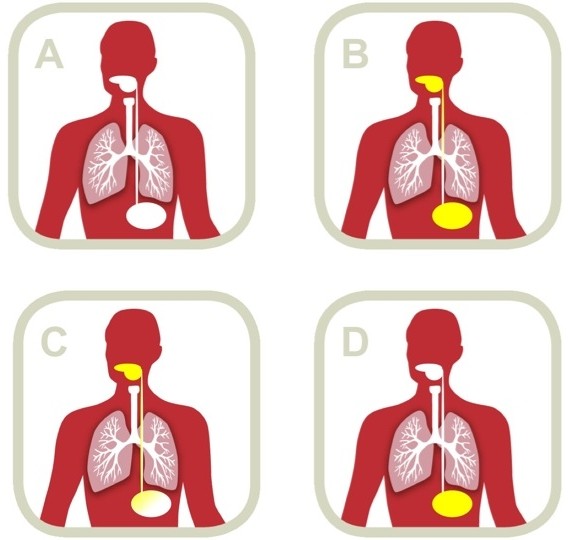
**Route of administration of prophylactic agents. **(A) no prophylaxis; (B) the concept of SDD, with the application of non-absorbable antibiotics in mouth and intestines; (C) the concept of SOD, with the application of non-absorbable antibiotics solely in the mouth (note that agents applied in the mouth could get into the stomach); (D) application of probiotics as in most trials in critically ill patients.

With SDD, non-absorbable antibiotics are administered in the mouth and intestines (and systemically, for the first few days after admission to the ICU); as such it should selectively eradicate of PPM in the oral cavity, throat, stomach and the intestines (Figure 1). With SOD, non-absorbable antibiotics are only administered in the mouth, and should selectively eradicate PPM in the oral cavity, and maybe throat, stomach and upper intestines, if (parts of the) non-absorbable antibiotics are swallowed. In only one study, probiotics were simultaneously administered in the mouth and the intestines [40]. Probiotics were administered solely to the stomach in the majority of the studies [35,36,38,39,41,42].

## Discussion

One conclusion that can be drawn from the retrieved randomized controlled trials of SDD in critically ill patients is that this strategy is an effective measure against VAP. Indeed, a vast majority of studies of SDD showed reduction of VAP rates with this strategy. SOD also seems an effective strategy against VAP. Notably, SDD and SOD were found equally efficient strategies with respect to prevention of mortality in critically ill patients. The preventive effects against VAP of probiotics are less certain. Additional studies are needed to confirm whether this strategy protects against VAP or not.

Although not all trials of SDD showed a beneficial effect, meta-analyses strongly suggested this prophylactic strategy to be a very effective measure against VAP [43-45]. Unfortunately, most studies of SDD were all too small to show any effect on mortality. Two recent well-powered randomized controlled trials of SDD, however, showed reduction of mortality of critically ill patients [53,62]. While these two trials did not report on reductions of VAP, it is suggestive that SDD lowered the incidence of this important complication. Interestingly, while the meta-analyses of trials of SOD showed no reduction of VAP [46], one of the two recently performed above mentioned trials showed also SOD to reduce mortality of critically ill patients [62].

While only four trials of probiotics showed benefits in critically ill patients, a recent meta-analysis suggested this prophylactic strategy to be an effective measure against VAP [48]. By contrast, one other meta-analysis of probiotics did not show benefits in critically ill patients [49]. Of note, after the publication of these two meta-analyses, three trials of probiotics have been published, two of them showed reduced incidences of VAP with probiotic therapy [40-42]. The differences between the two meta-analyses could be explained in different ways. First, one meta-analysis also included trials of post-operative patients who are often admitted to the ICU for too short a time to develop VAP [49]. Second, this meta-analysis did not include one important trial that showed reduced rates of VAP with probiotics [35].

Considering the rationale for antibiotics or probiotics as a preventive strategy against VAP, several remarks must be made. The suggestion that critical illness-associated infections are preceded by colonization of the digestive tract with PPM has never been adequately proven, let alone whether there is causality between colonization and infection. Furthermore, it is important to realize that the concept of colonization resistance has been demonstrated only in gnotobiotic mice (mice in which only certain known strains of bacteria and other microorganisms are present), and its relevance has never been documented in critically ill patients. Also, none of the beneficial effects of probiotics with respect to colonization prevention have been unequivocally demonstrated in critically ill patients. Further remarks include the fact that there are no studies that support the claim that a short course of systemic antibiotics prevents primary endogenous infections. Finally, while in the classical design of SDD it was claimed that secondary endogenous infections arise mostly from other patients via the hands of caregivers (necessitating the need for hand hygiene), this has never been supported by studies. Also, it is uncertain whether frequent surveillance cultures are needed to monitor the effectiveness of decontamination.

What should be noted is that almost all publications of trials of prophylactic antibiotics or probiotics lack a discussion on standard preventive measures against VAP. Such measures could include early weaning from mechanical ventilation, hand hygiene, aspiration precautions, and prevention of contamination, at times summarized with the acronym "WHAP" [63]. In a single-centre uncontrolled study it was demonstrated that an educational initiative on WHAP, directed at respiratory care practitioners and ICU nurses, was associated with decreases in VAP incidence rates of up to 61% [63]. Of course we should be careful in accepting results from single-centre uncontrolled studies with non-specific criteria for diagnosing VAP. However, it is suggestive that one problem with the interpretation of the reviewed trials of SDD, SOD and probiotics is that it is uncertain whether caregivers complied with other prevention strategies.

Although every literature review aims to find all studies addressing the question of the review, finding all studies is not always possible. It has been shown that those studies with significant results are easier to find than those without significant results. Also, studies with "positive" results are easier published than those with "negative" results. Over-representation of studies with significant results and "positive" studies in reviews may cause bias toward a positive result. We cannot exclude this to be the case in our review of antibiotics or probiotics against VAP.

It is yet unclear whether probiotics offer their benefits merely by preventing the colonization with PPM [64]. In one randomized controlled trial a decrease in the incidence of VAP was noted in patients receiving probiotics despite the fact that their colonization rates were left unaffected [39]. Another study showed that the administration of live *Lactobacillus *as opposed to killed *Lactobacillus *for the prevention of postoperative infections did not add any effect [65]. The mechanism of action of probiotics could be immunomodulatory more than non-immunologic (that is, by preventing colonization with PPM).

### Should we use SDD or SOD?

One recently published trial evaluated the effectiveness of SDD and SOD in a crossover study using cluster randomization in 13 ICUs in The Netherlands [62]. Mortality was the primary endpoint (while VAP was not an endpoint and not recorded). A total of 5,939 patients were enrolled in this trial: 1,990 assigned to standard care, 2,045 to SDD and 1,904 to SOD. Odds ratios for death in the SDD and SOD groups, as compared with the group of patients that received standard care, were 0.83, 95% confidence interval 0.72 to 0.97, and 0.86, 95% confidence interval 0.74 to 0.99, respectively. This study definitely supports the use of prophylactic antibiotics in critically ill patients. This study, however, also leaves us with a practical problem: Should we choose SDD or SOD? It is not realistic to consider a new trial that compares the effectiveness of SDD with SOD. Since there was only a small difference in effectiveness in this last trial, a new trial should include 10s of thousands of patients to show superiority of SDD over SOD, or vice versa.

Of course, one could (and should) consider the costs of each strategy: $12 for SDD and $1 for SOD [62]. And there is one other important issue that should be taken into consideration: SDD and SOD may differ in their risk of inducing antimicrobial resistance. Whether SDD or SOD are favorable with regard to development of antibiotic resistance is yet unknown. At present, a multicenter cross-over comparison study of SDD and SOD in ICU settings using either SDD or SOD for standard care is running in The Netherlands. Results from clinical and surveillance cultures will be used to assess development of antibiotic resistance in different pathogens.

### Should we use antibiotics or probiotics?

Prophylactic use may induce antimicrobial resistance. Many trials of SDD (and SOD) have been performed in The Netherlands, a country with low endemicity of resistant bacteria. Dutch settings, however, may not be representative for other settings. Without doubt, additional research is mandatory to determine whether SDD and SOD are safe strategies with respect to antimicrobial resistance in countries with higher endemicity of resistant pathogens.

Since probiotics are live bacteria, patients could become colonized and eventually develop probiotic-related infection. The currently available trials of probiotic therapy did not exhaustively address this issue, as they were not specifically designed for this outcome and were far too underpowered for that. Reports on VAP, endocarditis and bacteremia caused by probiotics [65-67], as well as a recently stopped trial of probiotics in pancreatitis patients because of increased mortality with probiotic treatment [47] suggest this scenario to be realistic [61].

It should be realized that studies of probiotics so far used different (combinations of) strains of live bacteria, sometimes combined with prebiotics. Each strain of probiotics may have additional, unique properties and actions towards specific targets. Present knowledge on these properties and actions, in particular in critically ill patients, is insufficient.

Furthermore, there is a need for further clarifications regarding doses, schedules and timing of probiotics for prevention of VAP and colonization, as to-date a great variability exists in the literature. Indeed, what should be noted is that in most trials probiotics were solely administered in the stomach. In only one trial the investigators applied probiotics simultaneously to the mouth and the intestines [40]. Interestingly, this trial showed the largest beneficial effect of probiotics. By contrast, with SDD antibiotics are administered in the mouth and intestines; with SOD antibiotics are administered exclusively in the mouth. It remains to be determined what route is superior for probiotics: both in the mouth (for oral eradication of PPM) *and *in the intestines (for intestinal eradication of PPM), or only in the intestines.

## Conclusions

SDD and SOD seem efficient preventive measures against VAP. SDD and SOD are equally effective with respect to the prevention of mortality. Future studies of SDD and SOD should address the issue of emerging resistance with increased antimicrobial pressure. Given the increasing antimicrobial resistance, probiotics deserve consideration in new trials. Such trials should be well-powered, and investigators should carefully consider where to administer the probiotics: in the mouth, in the intestines, or both. Finally, studies of probiotics in critically ill patients should have active surveillance for probiotic-induced diseases.

## Key messages

• SDD and SOD are efficient preventive measures against VAP and equally efficient strategies with respect to prevention of mortality in critically ill patients.

• The majority of trials of SDD/SOD did not exhaustively address the issue of emerging resistance, as most were not specifically designed for this outcome and were far too underpowered for that; use of SDD/SOD may be limited due to the risk of emerging resistance to the locally applied antibiotics.

• Trials of probiotic therapy did not adequately address the risk of colonization with probiotics and probiotic-related infection.

• Probiotic therapy deserves consideration in future trials.

• Trials of probiotic therapy should be well-powered, and investigators should carefully consider where to administer the probiotics.

## Abbreviations

ICU: intensive care unit; IPI: intrinsic pathogenicity index; PPM: potentially pathogenic microorganisms; SDD: selective decontamination of the digestive tract; SOD: selective oropharyngeal decontamination; VAP: ventilator-associated pneumonia.

## Competing interests

The authors declare that they have no competing interests.

## Authors' contributions

MJS was responsible for concept and design, analysis and interpretation of data, and critical revision of the manuscript for important intellectual content. LH was responsible for search of the literature, analysis and interpretation of data, and critical revision of the manuscript for important intellectual content. All authors read and approved the final manuscript.
